# Formation mechanisms of boron oxide films fabricated by large-area electron beam-induced deposition of trimethyl borate

**DOI:** 10.3762/bjnano.9.120

**Published:** 2018-04-24

**Authors:** Aiden A Martin, Philip J Depond

**Affiliations:** 1 Physical and Life Sciences Directorate, Lawrence Livermore National Laboratory, Livermore, California 94550, USA

**Keywords:** boron oxide, diffusion and growth, electron beam-induced deposition, surface reactions, trimethyl borate

## Abstract

Boron-containing materials are increasingly drawing interest for the use in electronics, optics, laser targets, neutron absorbers, and high-temperature and chemically resistant ceramics. In this article, the first investigation into the deposition of boron-based material via electron beam-induced deposition (EBID) is reported. Thin films were deposited using a novel, large-area EBID system that is shown to deposit material at rates comparable to conventional techniques such as laser-induced chemical vapor deposition. The deposition rate and stoichiometry of boron oxide fabricated by EBID using trimethyl borate (TMB) as precursor is found to be critically dependent on the substrate temperature. By comparing the deposition mechanisms of TMB to the conventional, alkoxide-based precursor tetraethyl orthosilicate it is revealed that ligand chemistry does not precisely predict the pathways leading to deposition of material via EBID. The results demonstrate the first boron-containing material deposited by the EBID process and the potential for EBID as a scalable fabrication technique that could have a transformative effect on the athermal deposition of materials.

## Introduction

Applications for boron-containing materials are diverse and include high-energy laser fusion targets [1], superconductors [2], X-ray optics [3], microelectromechanical systems [4], and neutron detectors [5]. All these alluring applications are, however, overshadowed by fabrication challenges at the microscale arising from instabilities produced during the thermally induced fabrication process. Exposure to high temperatures is incompatible with many substrate materials and leads to the development of undesirable stress, which often results in film cracking and delamination [1].

**Figure 1:**
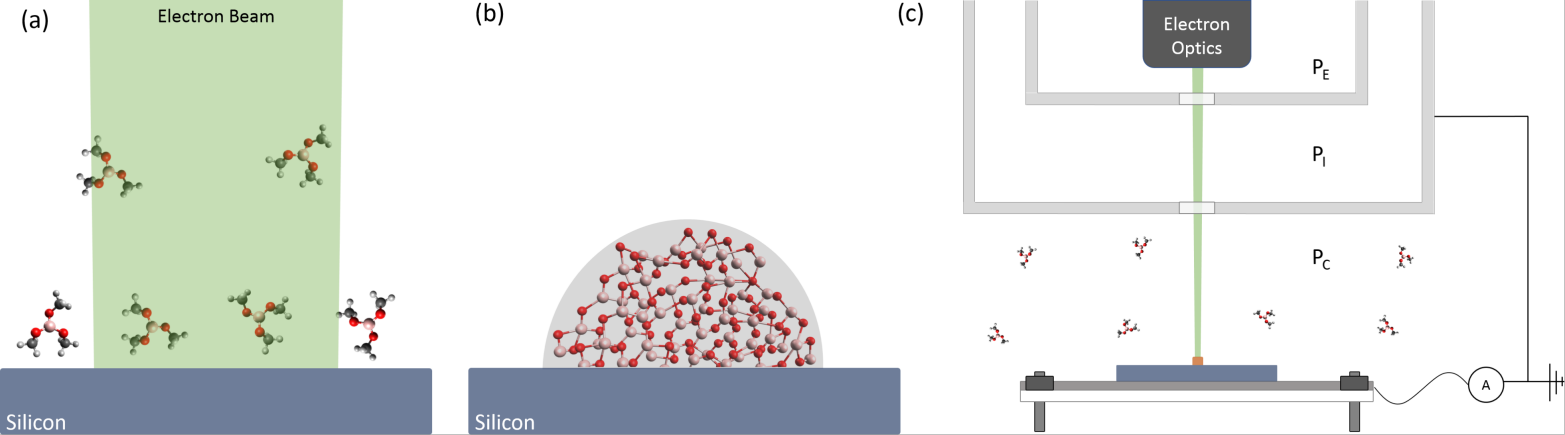
The EBID process and instrumentation. (a) Gaseous TMB precursor is admitted into the vacuum chamber where it adsorbs to the silicon substrate surface. (b) An electron beam dissociates adsorbed precursor molecules creating non-volatile fragments that bind to the surface forming a solid. (c) Schematic of the system using large-area EBID. The system comprises an electron flood gun, heater assembly, substrate current measurement circuitry, and three differentially pumped vacuum regions (P_E_, P_I_ and P_C_), which allows the electron optics to be maintained at high vacuum (ca. 10^−3^ Pa) while the pressure in the process chamber is of the order of 1 Pa.

A rapidly developing technique that has been demonstrated for a wide range of materials is electron beam-induced deposition (EBID) [6]. It avoids instabilities related to thermal- and mass-transport by overcoming the activation barrier for material deposition via electron-induced dissociation of surface-adsorbed precursor molecules into atomic or molecular fragments. The non-volatile fragments formed during this reaction bind to the substrate surface forming a solid (Figure 1a,b). EBID is typically performed in a high-vacuum chamber with the substrate at room temperature. During processing, a gaseous precursor is injected near the deposition site and undergoes subsequent reaction with an electron beam. The process has major advantages over thermal chemical vapor deposition (CVD) processes one of which being that the substrate is not exposed to the elevated temperatures required for the thermal decomposition of precursor molecules.

To date, the deposition of boron-containing materials through the EBID process has not been demonstrated. This is due to the majority of applications for EBID being targeted at the semiconductor industry, such as photolithography mask repair [7] and nanoscale fabrication of functional materials [8–9]. Materials of interest include carbon, silicon, various oxides, and a wide range of metals including gold, platinum and chromium (for a comprehensive list of materials and precursors please refer to the review by Utke et al. [10]). Conventional research has shown the efficient deposition of continuous films on the scale of nanometers to a few micrometers using modified scanning electron microscope (SEM) tools [10–11]. While deposition of films of the order of millimeters has been shown using residual contamination or diffusion pump oil in high-vacuum conditions [12–13], the deposition of films of this thickness under controlled gaseous environments similar to those found in nanoscale EBID processing have not been realized.

In this article, the deposition of boron-containing material through the EBID process is reported using trimethyl borate (TMB, B(CH_3_O)_3_) as precursor. TMB is an alkoxide-based molecule with structural properties similar to those of tetraethyl orthosilicate (TEOS, Si(C_2_H_5_O)_4_), which has been well characterized as a precursor for EBID of silica films [14]. TMB has previously been used for boron doping of SiO_2_ [15] and diamond [16], and the deposition of BCN fibres [17], BN nanotubes [18] and BN thin films [19–20]. Thin films were prepared using a novel, large-area deposition system designed for the directed deposition of macroscopic features using the EBID process. Deposition mechanisms of boron oxide from TMB precursor were determined from the thin-film deposition rate and stoichiometry as a function of the substrate temperature. The results reveal that high-purity boron oxide material is obtained at a substrate temperature of approximately 270 °C, and that the ligand type of the precursor molecule does not precisely predict the reaction pathway of the EBID process when compared to previous studies of TEOS. This knowledge is critical for optimization of precursor chemistry in other materials systems and for future modeling efforts.

## Experimental

EBID experiments were performed on a silicon substrate (100, n-type, P-doped, native oxide surface, MTI Corporation) with TMB precursor (99.95+%, Strem Chemicals, vapor pressure: 13.7 × 10^3^ Pa at 20 °C [21], thermally stable up to a minimum of 470 °C [22]) at various substrate temperatures using a custom-built, large-area deposition system (Figure 1c). The deposition system consists of an electron flood gun (Perkin-Elmer, Model 11-010), a differentially pumped vacuum system based on oil-free turbomolecular pump, a boron nitride heater assembly, substrate current measurement circuitry, a mass-flow controller and pressure sensors. The chamber consists of copper CF flanges and Swagelok VCR components for the precursor vacuum region except for the two O-rings used in the differential pumping apparatus. This provides a deposition rate attributed to residual contaminants of less than 50 nm in 30 min at room temperature under high vacuum (ca. 10^−4^ Pa). A focused, Gaussian profile 2 keV, 20 μA, 200 μm radius (ω_0_, radius at 1/*e*2 intensity) electron beam was used for the EBID experiments. The electron beam was steered onto the substrate at normal incidence through two 1000 μm diameter apertures, which restrict the gas flow into the differentially pumped electron source region. After pumping the chamber to a pressure of ca. 10^−4^ Pa, the sample was heated to 400 °C for 30 min and then TMB was introduced into the chamber and maintained at a pressure of 6.3 Pa. EBID was performed by irradiating an area of the substrate with a stationary electron beam for 20 min per deposit at a series of decreasing temperatures from 405 to 26 °C. The surface topography of the resulting deposits was measured by a Keyence VK-X100 laser scanning confocal microscope [23] (LCSM, specified height measurement resolution of 5 nm and measurement repeatability of 0.02 μm) and analyzed using the VK Analyzer software package (version 3.3.0.0). The chemical composition was determined by energy-dispersive X-ray spectroscopy (EDS) and scanning electron microscopy using a Phenom ProX SEM at 5 keV electron energy. The Phenom ProX SEM EDS subsystem consists of a silicon drift detector with a 25 mm^2^ active area and a resolution of ≤137 eV at Mn Kα, a Si_3_N_4_ ultrathin window, and a 10 eV per channel multi-channel analyzer. In situ optical images where captured using a Navitar Zoom 6000 Lens System and CCD Camera (Thorlabs, DCU224C). The optical images captured a region deposited at room temperature on a copper-coated silicon substrate (100 nm thick copper layer, MTI Corporation) under electron beam conditions identical to the temperature-dependent irradiations with a processing time of 30 min.

## Results and Discussion

**Figure 2:**
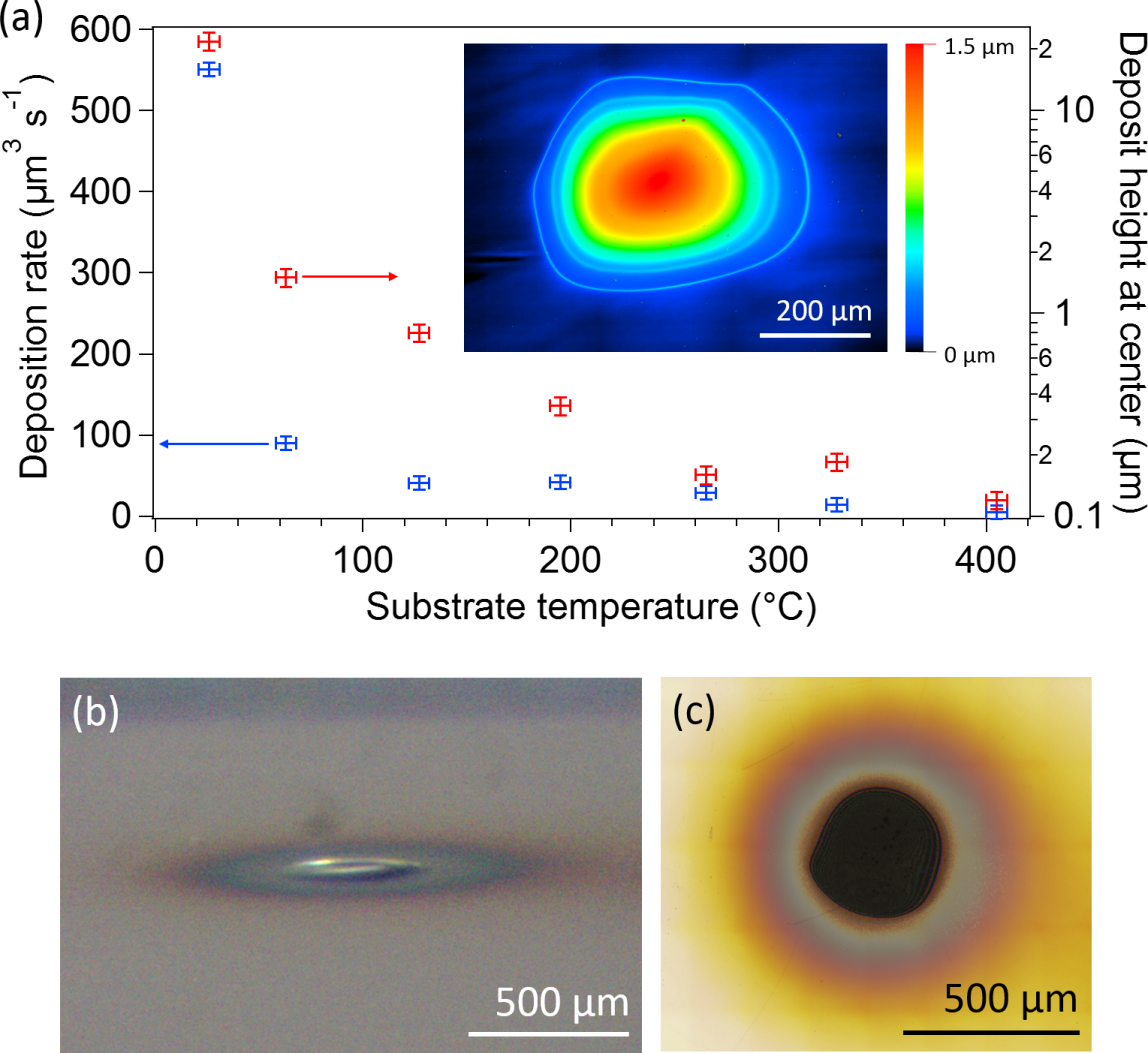
(a) Deposition rate of EBID using TMB precursor and a 2 keV, 20 μA Gaussian electron beam as a function of the silicon substrate temperature. Inset: LCSM height map of the deposit fabricated at a substrate temperature of 63 °C. (b) Side-on, in situ optical image of deposit produced by the large-area EBID system. The deposit was fabricated by performing EBID using TMB precursor on a room temperature copper-coated silicon substrate for 30 min. (c) LCSM optical image of the deposit shown in (b).

The volumetric deposition rate of the entire deposit and the height of the deposit at the center of electron beam as functions of the substrate temperature using TMB precursor are shown in Figure 2a. The material is rapidly deposited at room temperature and the rate sharply decreases with increasing substrate temperature. The sharp decrease in the rate of material deposition is caused by surface-coverage depletion of the precursor on the substrate at increasing temperatures due an increase in the thermal desorption rate [24]. At temperatures of 195 and 271 °C the majority of material is deposited in the outer periphery, approximately 100–150 μm from the electron beam center where replenishment of precursor proceeds predominantly through surface diffusion. LCSM images of all deposits are presented in Supporting Information File 1. Material deposited at 26 °C does not display any signs of cracking (Figure 2b,c), but is susceptible to laser-induced heating damage (see Raman spectroscopy discussion in Supporting Information File 1).

The decrease in deposition rate with temperature is expected for the majority of EBID precursors. However, it is in stark contrast to the silicon alkoxide precursor TEOS [14]. The deposition rate of TEOS recovers at high temperature through the activation of chemisorption where scission of Si–O bonds enables TEOS to react with the hydroxylated surface to form adsorbed ethoxysiloxanes [25–26]. TMB does not undergo the transition from physisorbed to predominantly chemisorbed reaction kinetics, because the surface bonding configuration and/or strength of the B–O bond does not allow for the activation of chemisorption in this system. This finding is significant as it confirms that alkoxy ligand precursors are not necessarily a perfect candidate for chemisorption, which would enable recovery of the deposition rate at high temperature.

When compared to conventional, EBID at the scale of a few micrometers at room temperature with TEOS [14] (estimated deposit height ca. 24 μm, 6.7 Pa precursor pressure, 25 keV electron energy, 80 electrons·Å^−2^·s^−1^ electron flux), the similarity of the deposition height profile of this study under comparable precursor and electron irritation conditions (maximum deposit height ca. 22 μm, 6.3 Pa precursor pressure, 2 keV electron energy, 32 electrons·Å^−2^·s^−1^ maximum electron flux) reveals that conventional descriptions of EBID deposition kinetics apply to these larger scales [27]. Therefore, recipes developed for nanoscale fabrication can be upscaled without the need for intensive optimization studies.

**Figure 3:**
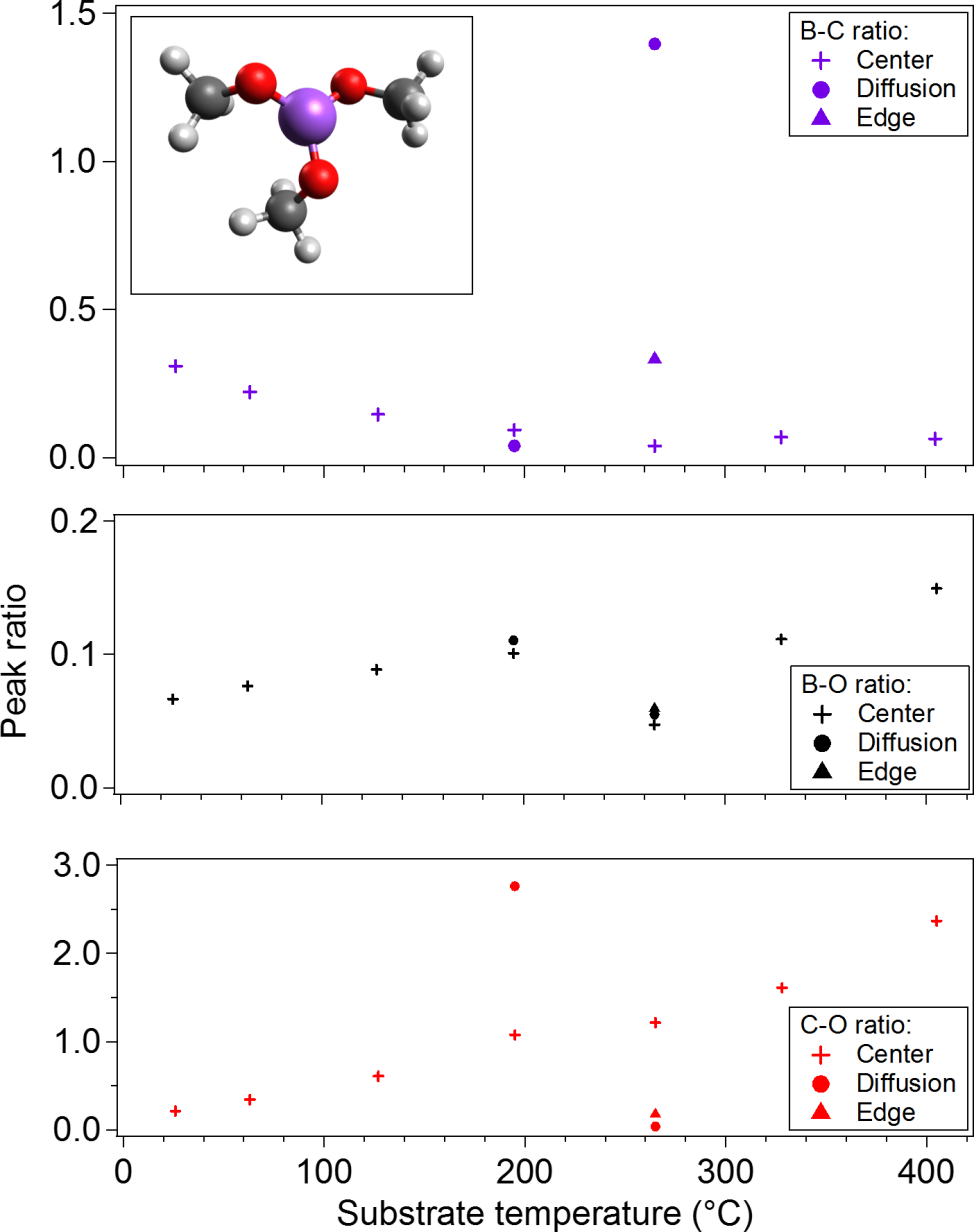
Elemental composition of material deposited by EBID using TMB precursor as a function of the substrate temperature. The composition is presented as the boron-to-carbon, boron-to-oxygen and carbon-to-oxygen Kα X-ray peak intensity ratios with no corrections applied. The composition was determined by EDS in the region at the center of the electron beam used for deposition, in the region of the deposit where an abrupt interface was formed due to the diffusion limited growth regime, and in the region at the outer edge of the deposit (ca. 50 μm from the diffusion region). These regions are labeled in Figure 4. Inset: TMB molecule.

EDS was performed to determine the composition of deposited material as a function of the substrate temperature (Figure 3). The 100 × 100 μm central region of each deposit was measured and analyzed to determine the boron-to-carbon, boron-to-oxygen and carbon-to-oxygen Kα X-ray peak intensity ratios. The concentration of boron and oxygen relative to carbon decreases with increasing substrate temperature in the central region. The increase in concentration of carbon at higher temperatures is ascribed to electron-induced dissociation of residual hydrocarbons that are present in the processing chamber or precursor material, which increasingly contribute to the EBID process when TMB undergoes rapid thermally stimulated desorption from the surface at higher temperature.

**Figure 4:**
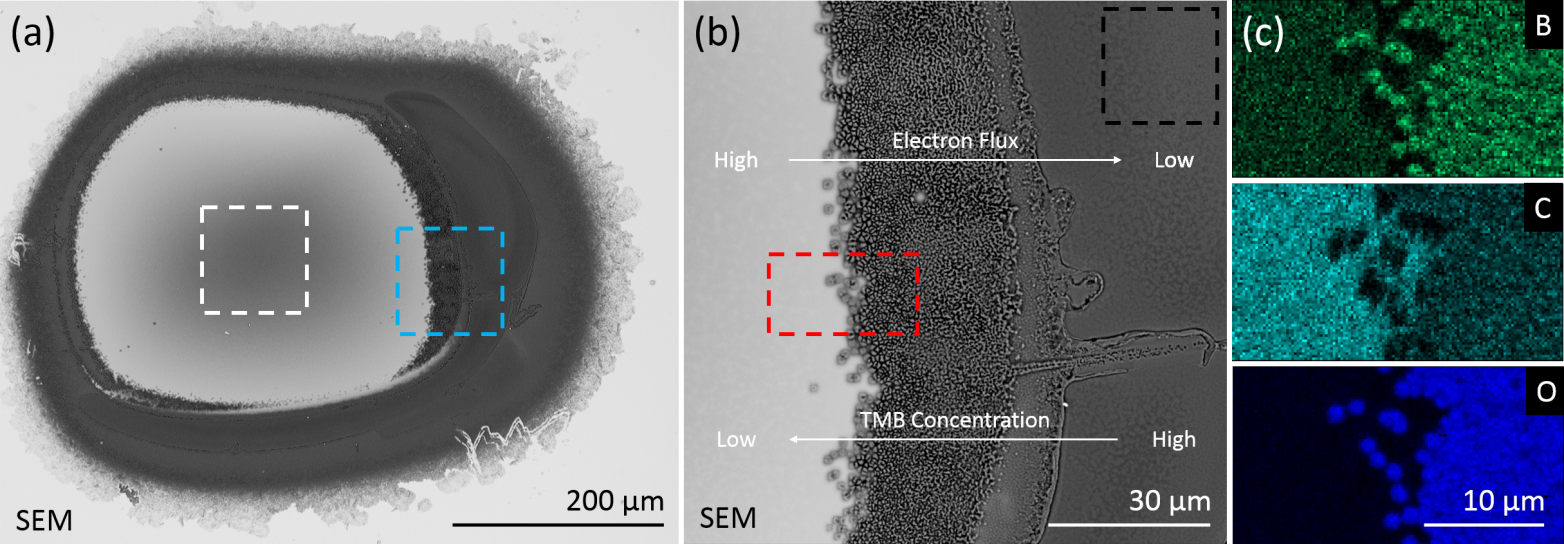
Backscattered scanning electron image of (a) the entire region and (b) the edge region (ca. 100 μm from the center of the electron beam, indicated by the blue box in (a)) of a deposit performed at a substrate temperature of 271 °C. At this temperature, diffusion-limited replenishment of TMB precursor results in an abrupt inner edge in the deposition profile. (c) Elemental maps of boron, carbon and oxygen at the TMB precursor depletion boundary measured by EDS (the region is indicated by the red box in (b)). Regions noted in Figure 3 as center, diffusion and edge are indicated by the white, red and black boxes, respectively.

The compound stoichiometry across the deposition profile was found to be temperature-dependent. The profile at 26 °C appears relatively uniform to the edge of the deposit (see Supporting Information File 1). At substrate temperatures of 195 and 271 °C an abrupt edge forms in the deposition profile at approximately 100 μm and 150 μm from the center of the electron beam respectively (Figure 4). This interface is formed by electron-induced dissociation of precursor that is diffusing across the concentration gradient between the outer periphery of the deposit and the depleted central region [28]. The composition of material at the diffusion interface in the deposit formed at 195 °C is carbon-rich compared to the center of the deposit, which is in stark contrast to the deposit formed at 271 °C. At 271 °C, the concentration of carbon relative to boron and oxygen decreases significantly at the diffusion interface, and the material at the interface was determined by an EDS standard-less ZAF correction method [29–30] to be BO_2.3_ with a small fraction of carbon impurity. The reported concentrations using the ZAF correction method were B: (28.4 ± 0.3)%, C: (5.9 ± 1.2)%, O: (65.7 ± 0.1)%.

The reduction in carbon content is similar to the behavior observed in TEOS, which on dissociation evolves hydrogen and ethylene from the surface [14,31]. Consideration of the electron flux-dependent stoichiometry of the deposits must be taken into account to confirm the temperature-dependent mechanism [32]. The electron flux in the regions where diffusion of precursor is the dominant replenishment mechanism at 195 and 271 °C is approximately 19 and 10 electrons·Å^−2^·s^−1^, respectively. The difference in electron flux for the results detailed here are small compared of the difference presented in the experiments by Höflich et al. where the electron flux difference between regions is approximately four orders of magnitude [32]. There is evidence for changes in the stoichiometry caused by the electron flux gradient in the 271 °C deposit. The region 200 μm from the center of the electron beam where the electron flux is approximately 4 electrons·Å^−2^·s^−1^ shows an increase in carbon content compared to the region at 150 μm, but this change is smaller than the difference between the diffusion mediated growth interfaces at 195 and 271 °C. With electron flux dependence excluded as the mechanism for changes in stoichiometry, the mechanisms of EBID using TMB precursor are ascribed to electron-induced dissociation of physisorbed precursor molecules exclusively, and temperature-dependent desorption of methyl ligands from dissociated TMB precursor, which result in large changes in the deposition rate and stoichiometry as a function of the temperature.

## Conclusion

In conclusion, the deposition mechanisms of EBID using TMB precursor were explored at silicon substrate temperatures between 26 and 405 °C. The deposition rate decreases rapidly with increasing substrate temperature, with TMB not undergoing activated chemisorption from the gas phase in stark contrast to the alkoxide-based silica precursor molecule TEOS. The morphology and composition of deposited material varied across the deposition profile at high temperatures. Material deposited from TMB precursor decreased in carbon content with increasing temperature revealing the activation of methyl ligand desorption. The results are interesting as they reveal the slight nuances in deposition mechanisms between the alkoxide-based systems TMB and TEOS, and therefore lead to the conclusion that ligand chemistry does not precisely predict EBID reaction kinetics. For the first time large-area, directed deposition by EBID was realized under gaseous precursor conditions analogous to those used for nanoscale processing. The electron flood gun based system enabled the deposition of boron-containing material with a minimum deposition cross-section of 500 μm in diameter. The results describe the first boron-containing material deposited via the EBID method and demonstrate a large-area system that extends the capabilities of EBID for material fabrication that can be applied to conventional precursor systems.

## Supporting Information

File 1 Additional experimental data
