# Supplementary material for: Formation mechanisms of boron oxide films fabricated by large-area electron beam-induced deposition of trimethyl borate
Source: Beilstein J Nanotechnol. 2018 Apr 24;9:1282–7. doi: 10.3762/bjnano.9.120 (PMC5942371; doi:10.3762/bjnano.9.120)
Supplement: File 1 — Additional experimental data [file Beilstein_J_Nanotechnol-09-1282-s001.pdf]

Supporting Information

for

Formation mechanisms of boron oxide films fabricated by large-area electron beam-induced deposition of trimethyl borate

Aiden A. Martin*[1] and Philip J. Depond[1]

Address: [1]Physical and Life Sciences Directorate, Lawrence Livermore National Laboratory, Livermore, California 94550, USA

Email: Aiden A. Martin - martin248@llnl.gov

* Corresponding author

Additional experimental data

Laser scanning confocal microscope images

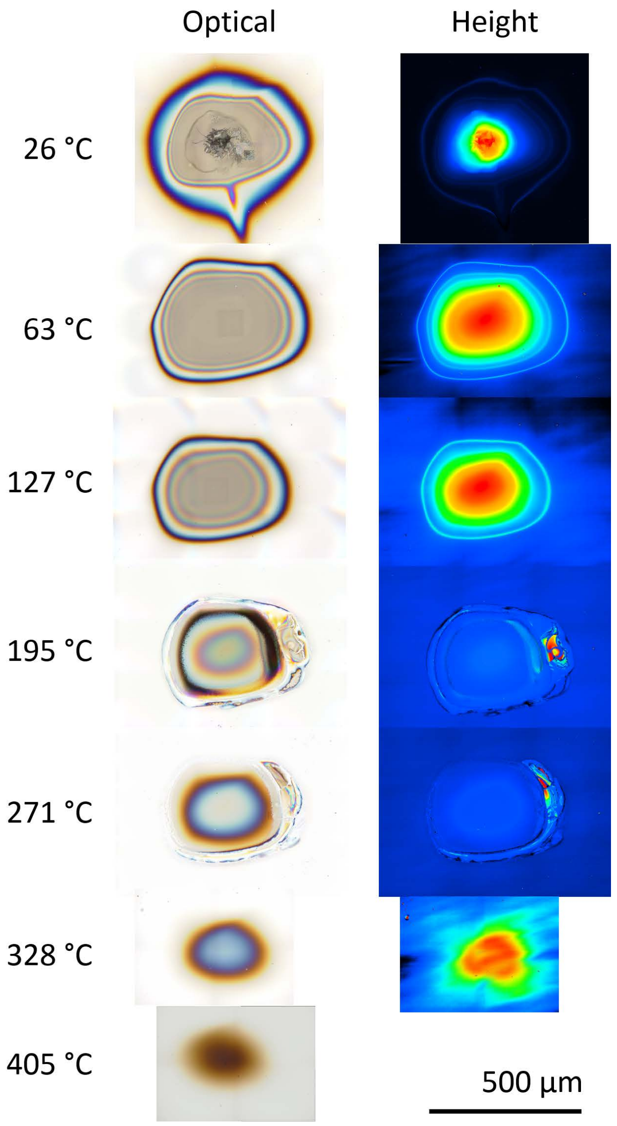

Figure S1: Optical images and height profiles measured by laser scanning confocal microscopy (LSCM) of material deposited by EBID using trimethyl borate (TMB) as a function of the substrate temperature. The height profiles are scaled for the minimum–maximum range at each respective temperature. The height profile for 405 °C is not shown as it is in the noise floor due to image stitching.

Raman spectroscopy

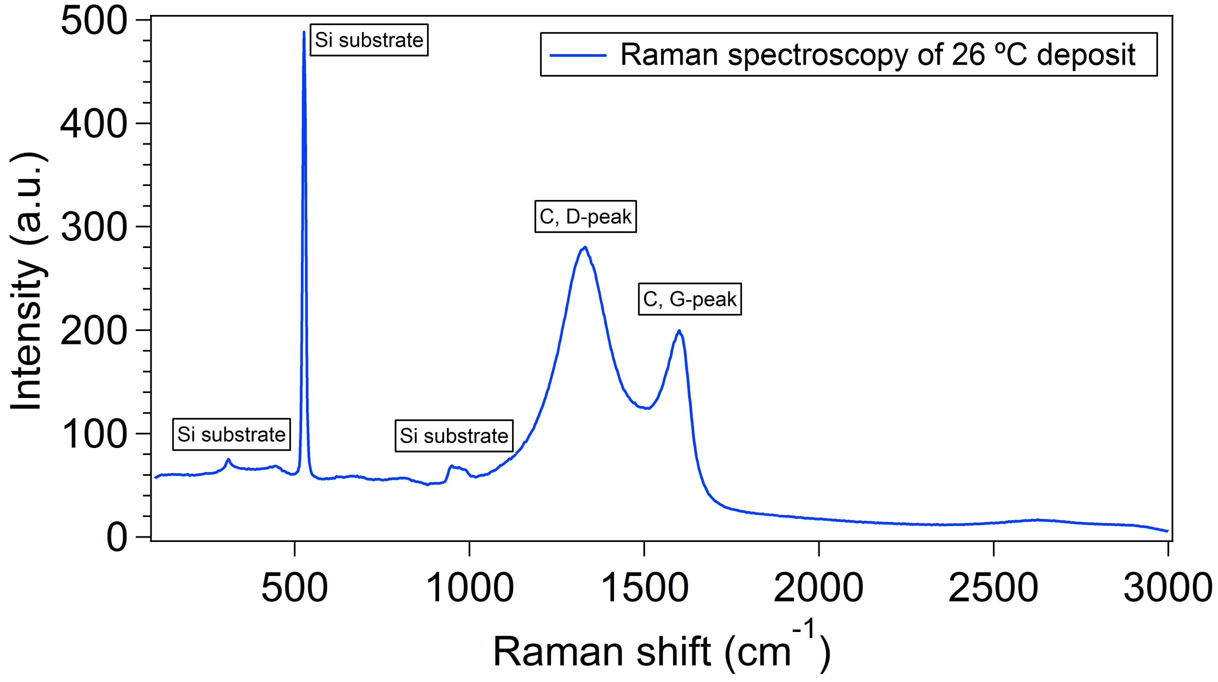

Figure S2: Raman spectroscopy measurement of material deposited by EBID using trimethyl borate (TMB) at a substrate temperature of 26 °C. The measurement was performed by using a Thermo Scientific Nicolet Almega XR micro-Raman system using a 633 nm, 1.67 mW laser and a 50× magnification, 0.5 NA objective lens. The Raman signature of the material corresponds to amorphous carbon. However, this measurement may not be representative of pristine material as the deposit is damaged by the laser during measurement. This damage is observed in optical and SEM images.

Energy-dispersive X-ray spectroscopy map

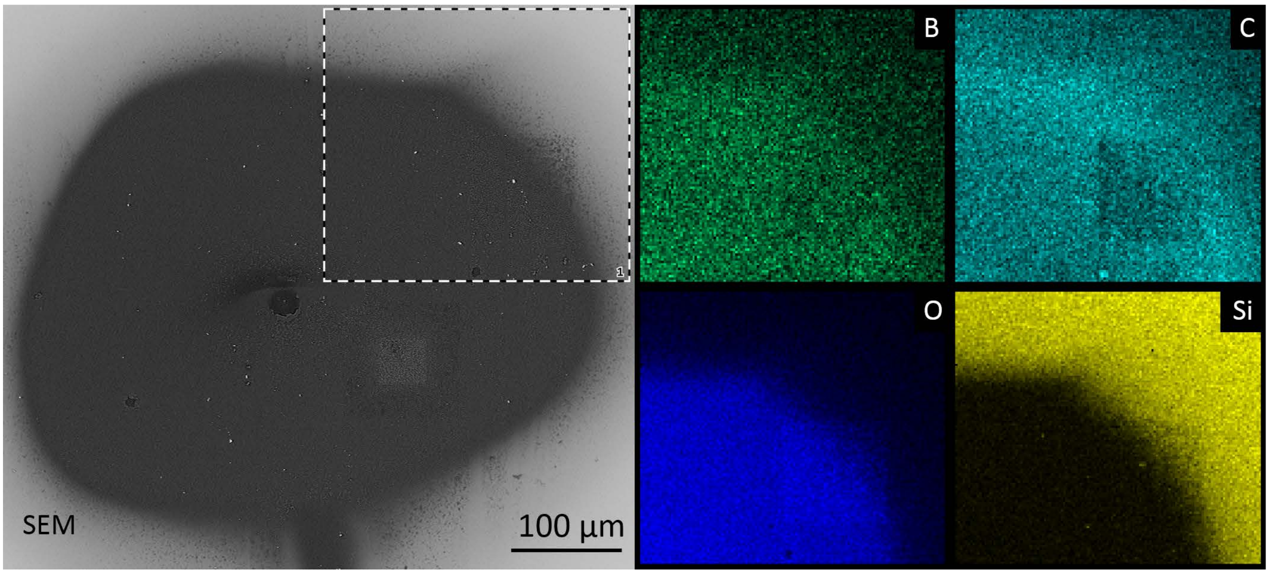

Figure S3: Elemental composition maps measured by energy-dispersive X-ray spectroscopy (EDS) of material deposited by EBID using trimethyl borate (TMB) at a substrate temperature of 26 °C. The maps were recorded using a Phenom ProX SEM at 5 keV electron beam energy. The elemental map was measured in the area indicated in the SEM image with boron, carbon, oxygen and silicon composition shown in each respective image.
